# An Enhanced Energy Balanced Data Transmission Protocol for Underwater Acoustic Sensor Networks

**DOI:** 10.3390/s16040487

**Published:** 2016-04-07

**Authors:** Nadeem Javaid, Mehreen Shah, Ashfaq Ahmad, Muhammad Imran, Majid Iqbal Khan, Athanasios V. Vasilakos

**Affiliations:** 1 COMSATS Institute of Information Technology, Islamabad 44000, Pakistan; ashfaqcomsats@gmail.com (A.A.); maji_iqbal@comsats.edu.pk (M.I.K.); 2 Allama Iqbal Open University, Islamabad 44000, Pakistan; smehreenshah@yahoo.com; 3 King Saud University, Riyadh 11451, Saudi Arabia; cimran@ksu.edu.sa; 4 Lulea University of Technology, Luleå 971 87, Sweden; vasilako@ath.forthnet.gr

**Keywords:** underwater acoustic sensor networks, routing protocol, network lifetime, energy consumption, throughput

## Abstract

This paper presents two new energy balanced routing protocols for Underwater Acoustic Sensor Networks (UASNs); Efficient and Balanced Energy consumption Technique (EBET) and Enhanced EBET (EEBET). The first proposed protocol avoids direct transmission over long distance to save sufficient amount of energy consumed in the routing process. The second protocol overcomes the deficiencies in both Balanced Transmission Mechanism (BTM) and EBET techniques. EBET selects relay node on the basis of optimal distance threshold which leads to network lifetime prolongation. The initial energy of each sensor node is divided into energy levels for balanced energy consumption. Selection of high energy level node within transmission range avoids long distance direct data transmission. The EEBET incorporates depth threshold to minimize the number of hops between source node and sink while eradicating backward data transmissions. The EBET technique balances energy consumption within successive ring sectors, while, EEBET balances energy consumption of the entire network. In EEBET, optimum number of energy levels are also calculated to further enhance the network lifetime. Effectiveness of the proposed schemes is validated through simulations where these are compared with two existing routing protocols in terms of network lifetime, transmission loss, and throughput. The simulations are conducted under different network radii and varied number of nodes.

## 1. Introduction

**Figure 1:**
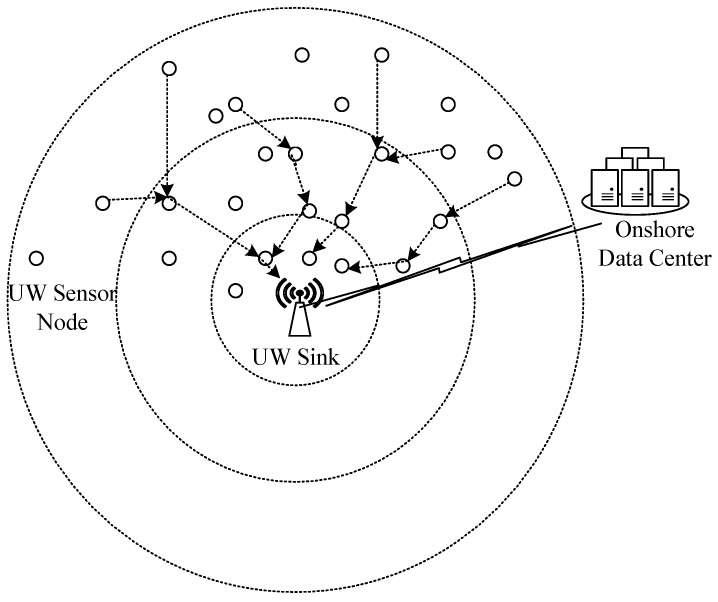
Underwater Acoustic Sensor Network (UASN) architecture.

Underwater Acoustic Sensor Networks (UASNs) are gaining popularity and attention due to their applications like oceanographic data collection, disaster prevention, tactical surveillance and maritime rescue [1]. UASNs consist of sensors and sink nodes that are deployed to perform collaborative monitoring tasks in a given area (refer Figure 1). The aquatic environment faces unique challenges like limited available bandwidth, multi-path fading, high bit error rate, fouling, corrosion and high end-to-end delay [2]. One of the major UASN challenges is limited available energy resources. Battery replacement in a harsh aqueous environment is difficult or almost impossible. Since the sensed data are routed towards sink, efficient and energy balanced routing mechanisms are needed to prolong network lifetime.

In UASNs, acoustic communication is preferred over radio communication because underwater environment hinders radio waves communication. The unique features of underwater environment do not allow the algorithms used in WSNs to be applied directly in UASNs. An acoustic modem’s energy consumption is quite different from typical radio transceiver [3]. Thus, either novel routing mechanisms or tailored ones are needed for UASNs that take into account the challenges of aquatic environment, and with the objective of network lifetime prolongation. In this regard, many routing protocols are proposed for UASNs [4,5,6,7,8]. Though these works increase the network lifetime, however, not to the extent that is needed.

Sensor nodes use one of the three ways to transmit data: direct, multi-hop and hybrid. In the first technique, nodes far away from sink die earlier due to high transmit power needed at longer distance. The second technique exhausts sensor nodes nearer to the sink due to more data transmission burden. The third one combines both direct and multi-hop transmission modes to somehow balance the load. Several existing works like [3,9,10] address energy balancing in both small and large-scale UASNs by exploiting the hybrid data transmission technique. Both [9,10] balance energy consumption in small-scale UASNs, however, their performance is worst in large-scale UASNs due to distant communication. Similarly, the protocols proposed in [3] are mainly designed for linear networks and perform well only under sparse network conditions.

Though the authors in [5,6,9], and [11] have contributed towards network lifetime prolongation (refer to Table 1), performance of these protocols degrades (in terms of lifetime, throughput and path loss) as the number of nodes are increased or the network area is increased. BTM’s performance degrades as the network area is increased because it is based on the direct communication mode. The transmit energy (in UASNs) is 100 times more expensive than receiving energy [12,13] which means that balanced use of the transmit energy among sensor nodes contributes to longer network functioning time as well as high throughput. Therefore, we focus on the efficient utilization of energy resources and propose two new routing protocols for UASNs; Efficient and Balanced Energy consumption Technique (EBET) and Enhanced EBET (EEBET). Both the techniques avoid long distance direct transmissions for balancing energy consumption. These protocols perform well under both sparse and dense network conditions. Both of the protocols initially establish communication links between sensor nodes on the basis of optimal distance threshold. For balanced energy consumption, initial energy of sensor nodes is divided into energy chunks termed as Energy Levels (RLs). After link establishment, nodes communicate with each other based on their energy levels. As the energy levels of communicating nodes differ, the predecessor node selects high residual energy level node within its vicinity as a next potential relay node. Consequently, the energy consumption of sensor nodes become balanced which is proved using Lemma 1. Furthermore, our techniques save the excessive transmit energy needed for direct data transmission over long distance. So, the technique improves network lifetime and is best suited for large scale UASNs. It is worth mentioning that this research work is an extended version of our previous work in [14].

The rest of the paper is organized as follows. Section 2 provides an overview of literature and the motivation behind this work. Section 3 illustrates the underwater energy consumption model. Section 4 explains detailed description of proposed schemes along with the mathematical model. In Section 5, performance evaluation is done through simulations and discussion of results is provided. Finally, overall work is concluded in Section 6.

## 2. Literature Review

As the underwater sensor nodes are powered by onboard batteries with limited capacities, energy efficient or energy balancing strategies are always demanded at all layers of the protocol stack. Since this work is limited in scope to the routing/network layer only, we discuss some of the recent work done subject to energy balancing in UASNs as follows.

Energy Balanced Hybrid data propagation (EBH) algorithm and Differential Initial Battery assignment (DIB) algorithm [3] consider linear UASN with hybrid transmission modes. In these algorithms, the sensor nodes switch between transmission modes according to their energy grades. The authors use differential battery assignment technique for different battery power levels. Similarly, authors in [18] proposed a multi-hop technique for UASNs subject to balanced energy consumption of the sensor nodes. In this technique, the downstream neighbors forward data packets as implicit ACKnowledgements (ACKs) for data packets that are sent a-priory. In [9], Cao *et al.* investigated Balanced Transmission Mechanism (BTM) in UASNs. In BTM, every sensor node selects transmission mode according to its energy level. Hence, every node utilizes energy in a balanced way and energy consumption in the network becomes somehow balanced. A dynamic addressing based routing technique for UASNs has been introduced by authors in [8]. This technique provides time-efficient and scalable routing without requiring any dimensional information.

The authors in [15] presented delay-sensitive and delay-insensitive distributed routing protocols for minimizing energy consumption of the sensor nodes. They have considered various application requirements along with the varying conditions of underwater channel. Based on the calling behavior of ultrasonic frog, a calling algorithm is proposed in [7]. The relay node selection process is based on the calling behavior of ultrasonic frog. In order to save energy, low residual energy and far away nodes choose sleep mode. In another work [16], the authors presented an energy efficient routing algorithm for UASNs based upon the residual energy of and distance between sensor nodes.

In [11], a Relative Distance-Based Forwarding routing protocol (RDBF) for UASNs has been proposed. RDBF calculates relative distance between sensor nodes and sink as a routing metric. Based on this routing metric, the forwarder nodes are selected. Moreover, the authors introduce a fitness factor to calculate holding time based on which the packets are forwarded. The authors claim that fitness function based packet forwarding mechanism results decreased probability of packet collision.

In [6], Depth-Based Routing protocol (DBR) is proposed for UASNs. In DBR, depth and holding time are used to forward the packets from source to destination, where the source lies at the bottom of the network field (high depth) and the destination resides at the surface of the network field (lowest depth). In [5], the authors improve DBR by choosing depth and residual energy based forwarder nodes (Energy-Efficient DBR (EEDBR) protocol). Mehmood *et al.* [17] extended DBR and EEDBR [5] to further improve the network lifetime. Their proposed technique limits the number of forwarder nodes using depth threshold parameter. Thus energy consumption is minimized.

**Counterpart Schemes in Brief:** In simulations (*i.e.*, Section 5), we have compared our proposed schemes with BTM [9] and UWDAR [19]. From the studied extensive state-of-the-art works, we choose BTM and UWDAR because their architecture and protocol operation is the closest to that of our work. Thus, we explain both these benchmark techniques in detail.

BTM is a hybrid data transmission routing protocol for UASNs. This protocol presents two algorithms; Efficient Routing Algorithm (ERA) and Data Balance Transmission (DBT) algorithm. Initially, ERA establishes tree between nodes which determines path for routing data packets from source to destination. Then, data transmissions occur as per DBT algorithm. Average number of nodes in a tree slice *i* are defined as 2i-1, where (2≤i≤N). BTM balances data load among sensor nodes by dividing the initial energy of each sensor node into energy chunks known as energy levels. A node transmits data to its one hop neighbor node till it depletes one energy level. The node which consumes one energy level earlier, starts direct data transmission towards sink till it again depletes one energy level. In this way, transmission mode is continuously shifted to achieve successive transmissions with balanced energy consumption costs. The proposed algorithms are implemented in both small and large scale UASNs, where radius considered for small scale UASNs is 0.2–1 km, while it is 1–5 km for large scale networks. However, BTM’s performance degrades as the network radius is increased.

Direct data transmissions are not favorable in network areas with very long network radii. They consume an abundance of energy to transmit data directly towards the sink, hence, network lifetime decreases. As a result, performance of this technique is very much degraded in large scale networks. BTM only balances energy consumption of sensor nodes within successive ring sectors, whereas, the difference of energy levels between near-to-the-sink nodes and the farthest nodes is notable. This difference increases with increase in transmission radius, thus, network lifetime decreases. Therefore, it is concluded here that this technique is impractical for large scale UASNs.

The other technique in comparison is the Data Aggregating Ring (DAR) technique [19]. Though this technique is proposed for terrestrial WSNs, we have implemented DAR using the distinctive underwater energy consumption model (in Section 3) for UASNs, hence named it UWDAR. This technique calculates hop-grades based on the number of hops a node is away from the sink. Single hop-grade nodes always directly transmit data to the sink during their in-charge as well as not in-charge time. All other network nodes (*i.e.*, nodes not in-charge hop grades), transmit data using multi-hop mode to in-charge hop-grade nodes, which further transmit it directly towards sink. Another contribution of this paper is the TDMA-like transmission scheduling mechanism defined to minimize collisions. Also, it helps sensor nodes to decide transmission delay with respect to their positions. A method is defined for determining in-charge time for nodes in different hop-grades. The major drawback of this technique is its backward data transmissions. Nodes far away also transmit data to in-charge hop-grade nodes even in-charge hop-grade nodes are at long distance from sink. This process consumes high energy which leads to short network lifetime. Sensor nodes switch between sleep and awake modes to save energy. This technique works for very small network radius, where direct data transmissions do not affect network performance.

**Figure 2:**
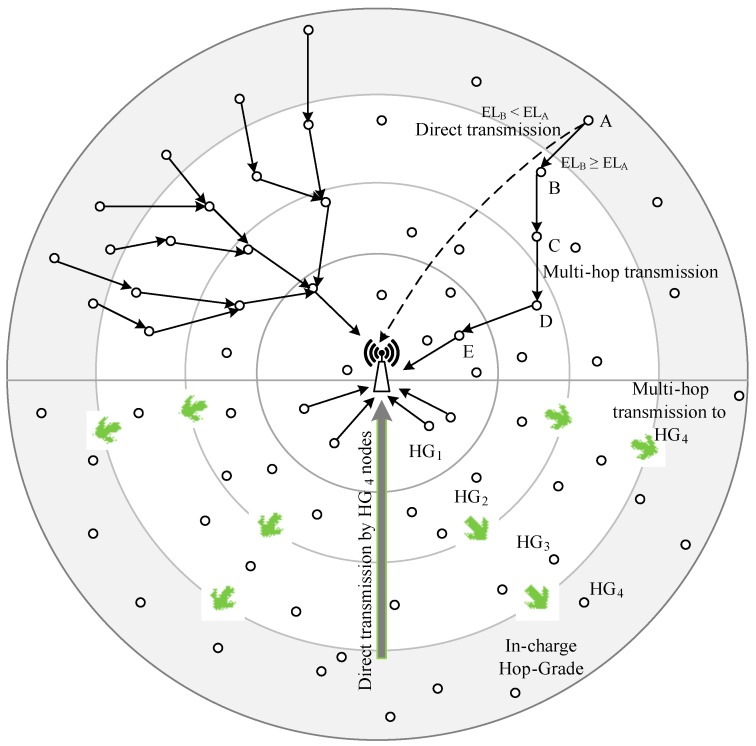
Data transmission in BTM and UWDAR.

Figure 2 illustrates the data transmission mechanism in BTM and UWDAR. As we can see in the figure, BTM adopts hybrid transmission mechanism and switches between multi-hop and direct transmission mode on the basis of difference in energy levels. While UWDAR assigns in-charge time to each hop-grade periodically according to their traffic characteristics, where, in-charge hop-grade is responsible to collect data from all network nodes and transmit it directly to the sink. This technique works for a limited geographical region due to its backward data transmission constraint.

## 3. Underwater Energy Consumption Model

The underwater communication is influenced by many factors like propagation delay, operating frequency, bandwidth, path loss, and transmission loss. Thus, energy consumption model designed for acoustic communication must consider all these factors. The attenuation at distance *d* for a signal with frequency *f* in an underwater acoustic channel is calculated as,

(1) $$A\left(d,f\right)=d^{k}\upsilon^{d}$$

where *k* denotes the spreading factor and *υ* is the absorption coefficient expressed in dB/km. The spreading factor *k* specifies the signal’s propagation geometry and its value varies for different energy spreading types. The value of *k* for spherical spreading is 2 while cylindrical spreading commonly uses k=1 and its practical value is 1.5. The absorption coefficient is based on the signal frequency measured in kHz. We can calculate absorption coefficient as [20],

(2) $$\upsilon =10^{\alpha (f)/10}$$

(3) $$\alpha \left(f\right)=\frac{0.11f^{2}}{1+f^{2}}+\frac{44f^{2}}{4100}+f^{2}+2.75\times 10^{-4}f^{2}+0.003$$

The above formula is valid for frequencies above few hundred Hertz. Whereas, the following formula is valid for lower frequency ranges.

(4) $$\alpha \left(f\right)=0.002+\frac{0.11f^{2}}{1+f^{2}}+0.011f^{2}$$

The power consumption $P_{t}$ to transmit data over distance *d* is given below,

(5) $$P_{t}=P_{o}A\left(d,f\right)$$

A sensor node consumes energy (denoted as $R_{Tx}\left(x,d\right)$) to transmit *x* bits packet over distance *d*.

(6) $$R_{Tx}\left(x,d\right)=xP_{o}d^{k}\upsilon^{d}t$$

where *t* is the transmission duration measured in seconds. Similarly, to receive a data packet a node consumes energy calculated as follows,

(7) $$R_{rx}\left(x\right)=xP_{r}t$$

where $P_{r}$ depends on the receiver and is a constant term.

## 4. EBET and EEBET: Proposed Techniques

Provided a target network area, the sensor nodes are deployed in a random-uniform way and the sink node is placed at the center of the network field. By random uniform we mean; (i) same number of nodes are deployed in each ring sector; and (ii) the deployment of nodes within a given ring sector is random. The assumptions and descriptions are as follows.

- All sensor nodes are homogeneous with limited battery power and are location-aware using existing positioning method (Received Signal Strength Indicator (RSSI) as location finder [21], and MoteTrack location identification scheme [22]). By location aware we mean that each sensor node knows its location.
- The sensor nodes always have data to send.
- The data reporting mechanism is periodic.
- The static sink resides on the surface of water.

**Figure 3:**
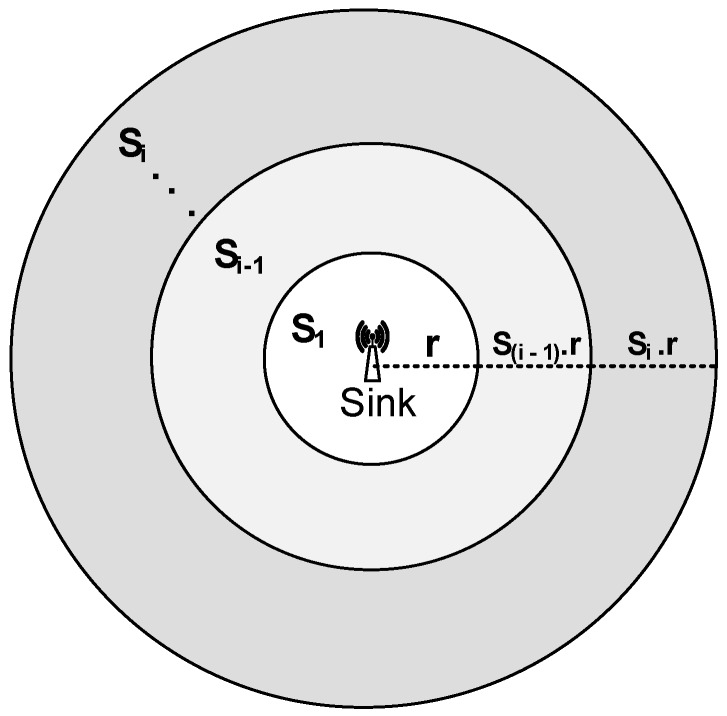
Network field division into ring sectors.

Location awareness (Equations (8) and (10) are used to find the neighbors of a node. Once the neighbors are identified, suitable relays are needed to forward the data from source to destination. In order to select/choose the relays, the network area is divided into regions known as Ring Sectors Sr. The maximum number of ring sectors $Sr_{max}$ depends on the network radius *R* and the optimum transmission distance threshold $O_{t}$. Relation between *R* and $O_{t}$ can be found from Equations (13) and (16) in [9]. Using these two equations, we find $O_{t}$ for different *R*. Using values of $O_{t}$, we find $Sr_{max}=\frac{R}{O_{t}}$. The slice containing sink is denoted by $Sr_{1}$ with radius *r*. Next to $Sr_{i-1}$ is $Sr_{i}$ where $i=1-Sr_{max}$ with radius (i-1)×r and i×r, respectively, as shown in Figure 3. The total number of nodes in each Ring Sector is *n* such that the total number of nodes in the network is *N* ($N=Sr_{max}\times n$).

### 4.1. Protocol Operation

The following sub-sections provide detailed functioning of our proposed schemes; EBET and EEBET.

#### 4.1.1. EBET

The key insight of this technique is to achieve a dual objective; energy-efficiency and balanced energy consumption. Data transmission occurs either by multi-hopping or by selecting suitable high RL relay node to transmit data to the sink. The core design of EBET is the use of high RL node as a relay node when the residual RLs of two successive nodes differ.

The EBET works in two phases; route establishment phase and data transmission phase. After network configuration, suitable forwarder nodes are selected by route establishment phase. Then the nodes communicate during data transmission phase.

##### Route Establishment

**Figure 4:**
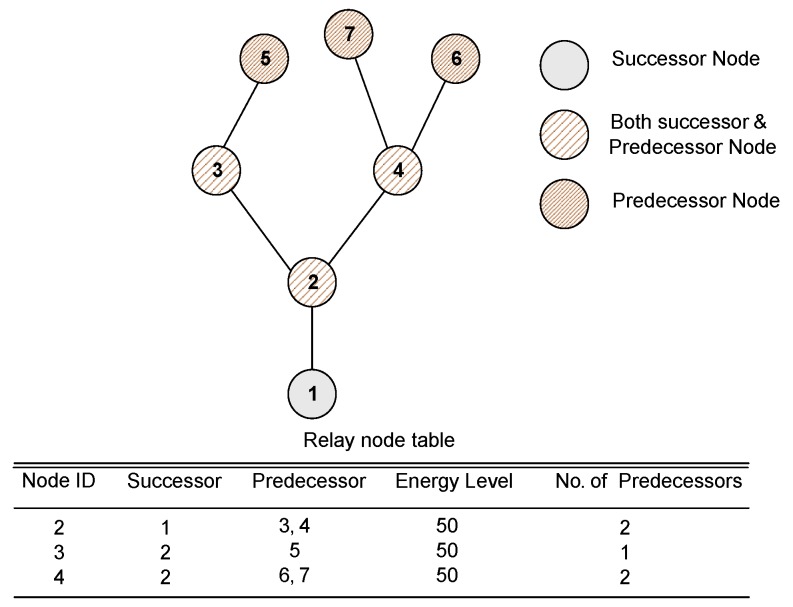
Successor and predecessor nodes with exemplary relay node table.

**Figure 5:**
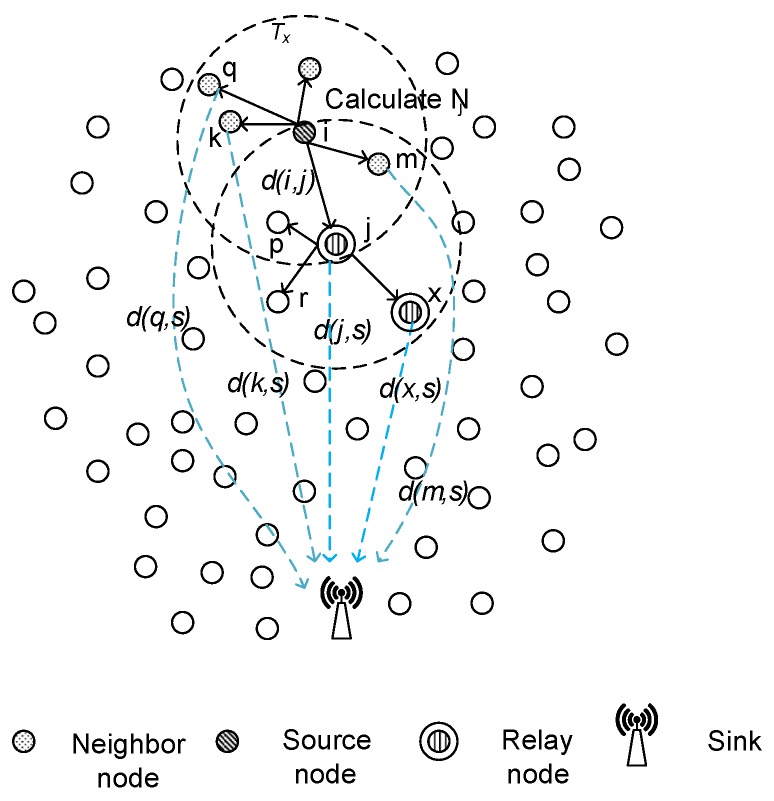
Relay node selection using $N_{j}$ calculation.

During the route establishment phase of EBET, sensor nodes look for suitable forwarder nodes. The sensor nodes form links with each other based on the value of optimum distance threshold $\left(O_{t}\right)$. We assume that the sensor nodes and the sink are location aware, *i.e.*, each node knows its own location. Every node exchanges its location information along with the coordinate position of sink to all the nodes in their transmission range. Each receiving node finds its neighbors using neighbour cost parameter $N_{j}$ [9] as follows:

(8) $$N_{j}=\alpha \left|d\left(i,j\right)-O_{t}\right|+\left(1-\alpha \right)d\left(j,s\right)$$

where *α* is a system parameter set as 0.5, d(i,j) is the distance between two nodes namely *i* (sending node) and *j* (receiving node), and d(j,s) is the distance between node *j* and sink *s*. The optimum threshold values (given in Table 2) set for node’s transmission distance are calculated using [10]. Node with minimum $N_{j}$ value is selected as a relay node. It is worth mentioning that $N_{j}$ information is maintained in routing table of predecessor node as shown in Figure 4 such that its calculation helps a sensor node to select a relay node at optimum distance nearer to sink. Node with minimum $N_{j}$ value informs its successor about its nomination as a relay node along with the number of nodes in its routing table containing $N_{j}$ values. This information is sent to help a relay node to check the limit imposed on the number of packets it can forward. A node can forward maximum three data packets at a time. This is due to the limitation on the number of neighbors of a node. Each node maintains maximum two neighbors at a time. We imposed this limit to reduce burden of nodes. If we allow each node to have maximum neighbors, their energy levels start decreasing sharply and nearer nodes die within no time, thus, network lifetime shortens. Also, this limitation helps in balancing energy consumption in the network. The same process continues till the node has sink in its optimum transmission distance. The node with sink in its transmission range directly transmits data to sink without further relaying. Node sending query packet is the predecessor node while receiving node (potential relay node) is called successor node for the sender. Table 2 shows optimum distance against network radius R. We show EBET in Algorithm 1. Figure 5 shows the selection of relay node by calculating $N_{j}$ and selects a node having minimum $N_{j}$ value.

##### Data Transmission

A set of routes are obtained during route establishment phase. Therefore, data communication occurs over the same links between nodes during data transmission phase. The transmit energy is 100 times more expensive mainly for underwater communication. For balanced energy consumption, we have adopted the concept of RLs.

Let the initial energy of each node be $E_{o}$. The $E_{o}$ of each sensor node is divided into Energy Levels RLs. Equation (9) calculates the value of Unit Energy Level (URL), 0<URL<1,

(9) $$URL=\frac{E_{o}}{L}$$

where *L* is the optimum number of RLs which are to be calculated in this section. The total energy consumption of a node includes sensing energy $R_{sen}$, receiving energy $R_{rx}$ and the transmit energy $R_{Tx}$. To calculate the total energy consumption $R_{t}$ of source *i* and receiver node *j* at time *t*, we have following equation.

(10) $$\begin{array}{c} R_{t}^{i}\left(t\right)=R_{sen}\left(x\right)+R_{rx}\left(x\right)+R_{Tx}\left(x,d\right)\;\;where\;\;\;i\in Sr_{i} \end{array}$$

When the data transmission process starts all the network nodes in different Ring Sectors have same Residual RLs, $R_{RL}$ as shown in Equations (11) and (12). Every node transmits data to its next hop neighbor (successor) using multi-hop method; $R_{RL}^{j}$ and $R_{RL}^{k}$ denote $R_{RL}$ for $j\in Sr_{i}$ and $R_{RL}$ for $k\in Sr_{i-1}$.

(11) $$R_{RL}^{j}=\frac{E_{o}}{\beta }\;\;\;\;\;\;\;where\;j\in Sr_{i}$$

(12) $$R_{RL}^{k}=\frac{E_{o}}{\beta }\;\;\;\;\;\;\;where\;k\in Sr_{i-1}$$

**Figure 6:**
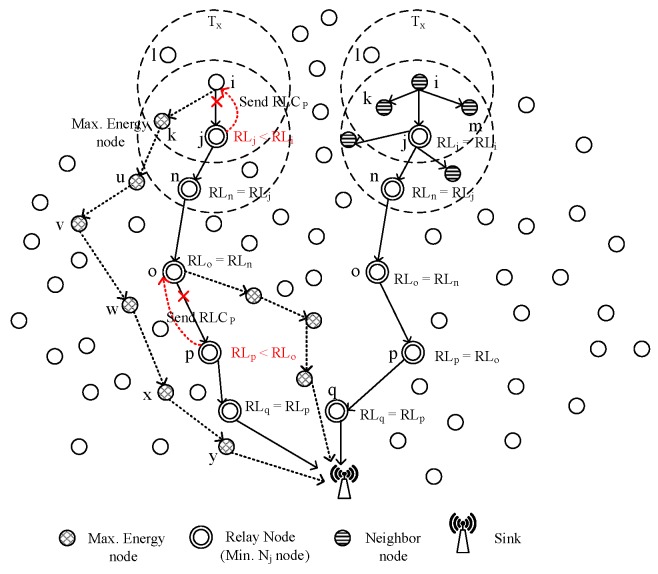
Variations in relay node selection based on energy levels.

Figure 6 shows the selection of relay node with variations in energy levels. As the nodes send data only to their next-hop node within their optimum transmission range. Here we use URL = *β*. We assume $R_{Tx}\left(x,d\right)\leq \beta$. Now we calculate the $R_{RL}$ of sensor node ($j\in Sr_{i}$) at time $t^{\prime }$, *i.e.*, $R_{RL^{\prime }}^{j}$ (the time when $R_{RL}^{k}<R_{RL}^{j}$).

(13) $$R_{RL^{\prime }}^{j}=\frac{E_{o}}{\beta }-\frac{\beta }{3}\;\;\;\;\;\forall \left(j\in Sr_{i}\right)$$

(14) $$R_{RL^{\prime }}^{k}=\frac{E_{o}}{\beta }-\beta \;\;\;\;\;\;\;\;\forall \left(k\in Sr_{i-1}\right)$$

As the process evolves, the nodes in nearer regions to the sink consumes URL earlier than the far away nodes due to additional relaying of data packets. We assume that a node can transmit data of two more nodes along with its own data. Equations (13) and (14) relates our assumption and specifies the decrease in Energy Level of a node. As the node *k* belongs to $Sr_{i-1}$ which is nearer to the sink than the Ring Sector $Sr_{i}$, therefore, its URL drops and the decrease in energy level of node *j* is its one third. Before sending next data packet, node *k* checks for the following condition:

(15) $$R_{RL}^{k}<R_{RL}^{j}$$

If the condition specified in Equation (15) is true, then the node informs its predecessor node by sending a control packet to stop transmitting its data to that node. Thus the node only transmits its own data to next hop neighbor node. This approach avoids the introduction of critical nodes in the network and hence prevents the network from partitioning [23]. However, this approach consumes surplus energy which leads to reduced network lifetime. The predecessor then looks for another relay node to restart its data transmission. It selects a node having high energy level than itself within its transmission range. In this way, the chain of linked nodes break into multiple chains and more data packets simultaneously arrive at sink. Also, energy consumption is balanced by dividing the load of data packets over multiple nodes. We opt to choose direct transmission over long distance. However, it consumes more than one Energy Level in transmitting data over long distance which decreases network lifetime. Lemma 1 proves the energy balancing in EBET by specifying the Energy Level difference between two nodes in two consecutive Ring Sectors is not more than URL.

**Lemma 1.**  *If* $R_{Tx}\left(x,d\right)<\beta$ *then* $RL_{j}-RL_{k}\leq URL$, *where* $\left(j\in Sr_{i}\right)$ *and* $\left(k\in Sr_{i-1}\right)$

**Proof:**  As $R_{Tx}\left(x,d\right)<RL$, then in sending one data packet a node consumes less than unit Energy Level. Using Equations (11)–(14), we have:

(16) $$\left(R_{RL}^{j}+R_{RL^{\prime }}^{j}\right)-\left(R_{RL}^{k}+R_{RL^{\prime }}^{k}\right)\leq \beta$$

(17) $$\left(\frac{Eo}{\beta }+\left(\frac{Eo}{\beta }-\frac{\beta }{3}\right)\right)-\left(\frac{Eo}{\beta }+\left(\frac{Eo}{\beta }-\beta \right)\right)\leq \beta$$

(18) $$\frac{2E_{o}}{\beta }-\frac{\beta }{3}-\frac{2E_{o}}{\beta }+\beta \leq \beta$$

(19) $$\beta -\frac{\beta }{3}\leq \beta$$

Hence, the difference of RLs between any two nodes in successive Ring Sectors is not more than URL. By using Lemma 1, we calculate max. residual energy of a node in $Sr_{i}$ as

(20) $$R_{e}=\left(i-1\right)\frac{E_{o}}{L}\;\;\;\;\;\;\;where\;i=1-Sr_{max}$$

Therefore, the total remaining energy of all nodes is calculated as follows,

(21) $$E_{res}^{Net}=\sum_{i=2}^{Sr_{max}}\frac{E_{o}}{L}\left(i-1\right)2^{\left(i-1\right)}$$

In the above equation, $2^{\left(i-1\right)}$ is the maximum number of nodes in each layer of the tree and (i-1) specifies that the maximum RL difference between nodes in successive Ring Sectors is 1. As we assume that the complete energy depletion of first sensor node collapses network. Therefore, the energy consumption in sending control packets by all the network nodes is as follows,

(22) $$E_{c}^{Net}=\sum_{i=1}^{Sr_{max}-1}LP_{o}d^{k}a^{d}t\times 2^{\left(i-1\right)}$$

**Theorem 1.**  *If* $R_{Tx}\left(x,d\right)\leq \beta$, *we can calculate optimal Energy Levels as:*

(23) $$L=\sqrt{\frac{E_{o}\left(2^{Sr_{max}}.Sr_{max}-2\left(2^{Sr_{max}}-1\right)\right)}{P_{o}d^{k}a^{d}t\left(2^{Sr_{max}-1}-1\right)}}$$

**Proof:**  We calculate the total energy wasted in the network using Equations (22) and (23)

(24) $$E_{w}^{Net}=E_{res}^{Net}+E_{c}^{Net}$$

(25) $$E_{w}^{Net}=\sum_{i=2}^{Sr_{max}}\frac{E_{o}}{L}\left(i-1\right)2^{\left(i-1\right)}+\sum_{i=1}^{Sr_{max}-1}LP_{o}d^{k}a^{d}t2^{\left(i-1\right)}$$

Taking derivative of Equation (22) with respect to *L*, we have Equation (26).

(26) $$E_{w^{\prime }}^{Net}=\frac{-E_{o}}{L^{2}}\sum_{i=2}^{Sr_{max}}\left(i-1\right)2^{\left(i-1\right)}+P_{o}d^{k}a^{d}t\sum_{i=1}^{Sr_{max}-1}2^{\left(i-1\right)}$$

(27) $$-E_{o}\sum_{i=2}^{Sr_{max}}\left(i-1\right)2^{\left(i-1\right)}+P_{o}d^{k}a^{d}tL^{2}\sum_{i=1}^{Sr_{max}-1}2^{\left(i-1\right)}=0$$

(28) $$L^{2}=\frac{E_{o}\sum_{i=2}^{Sr_{max}}\left(i-1\right)2^{\left(i-1\right)}}{P_{o}d^{k}a^{d}tL^{2}\sum_{i=1}^{Sr_{max}-1}2^{\left(i-1\right)}}$$

Thus, we obtain optimum number of Energy Levels as follows,

(29) $$L=\sqrt{\frac{E_{o}\left(2^{Sr_{max}}\cdot Sr_{max}-2\left(2^{Sr_{max}}-1\right)\right)}{P_{o}d^{k}a^{d}t\left(2^{Sr_{max}-1}-1\right)}}$$

The optimum RLs depend on the initial energy of sensor nodes $E_{o}$, transmit power $P_{o}$ and the Ring Sector numbers $Sr_{max}$.

Algorithm 1 shows the pseudo-code of EBET, where, $EL_{s}$ and $EL_{p}$ are energy levels of successor and predecessor, respectively.

**Algorithm 1:**: EBET1:**procedure:** INITIALIZATION. 2:TotalEnergyLevels ← m3:UnitEnergyLevel ← $E_{o}/m$4:RelayType ←*μ*5:return TRUE6:end procedure7:**procedure:** NEIGHBORQUERYRECEIVED8:*λ* ← d(i,j)9:*ς* ← d(j,s)10:$N_{j}\leftarrow \alpha \left|\lambda -O_{t}\right|+\alpha \varsigma$11:*ψ* ← $min(N_{j})$12:SendNeighborFoundAck.Id = IdOfRelayNode13:SendNeighborFoundAck()14:return TRUE15:end procedure16:**procedure:** OneEnergyLevelConsumed17:ControlMessageSend()18:**if** $EL_{s}<EL_{p}$ **then**19: $SendNeighborFindingMessage.EL=EL_{i}$20: SendNeighborFindingMessage()21: NeighborFoundAck = IdOfHighRLNode22: RelayType = *σ*23:**end** **if**24:end procedure25:**if** $EL_{s}>=EL_{p}$ **then**26: RelayType = *μ*27: end procedure28:**end** **if**

#### 4.1.2. EEBET

In EEBET, network field is divided into sub areas called Ring Sectors. The number of nodes in each ring sector varies according to its data load and the distance from the sink. For example, if for a given *R*, $Sr_{max}=4$. Then, the ring sectors $R_{1}$, $R_{2}$ and $R_{4}$ have same number of nodes while $R_{3}$ has more nodes. This is because the nodes in $R_{3}$ have increased data load and their distance from sink is greater than that of $R_{1}$ and $R_{2}$ nodes, respectively. $R_{1}$ and $R_{2}$ have same number of nodes because these are not engaged in reception or relaying of other nodes’ data. On the other hand, $R_{4}$ nodes have the data load of $R_{2}$ nodes, however, their distance from sink is less than that of $R_{3}$ nodes. Thus, we kept same number of nodes in $R_{1}$, $R_{2}$ and $R_{4}$. Whereas, $R_{3}$ has relatively high number of nodes. The sink node is deployed on the surface of water. All the sensor nodes transmit data to the sink which transmit it to the onshore data center. Nodes can vary their transmission range accordingly. EEBET search for suitable forwarder node during route establishment and transmit data to that node in data transmission phase. The EEBET addresses the limitations of both BTM and EBET techniques. Detailed operation of EEBET is as follows.

##### Route Establishment

The EEBET more intelligently selects the relay node during route setup phase than the BTM and the EBET techniques. One of the major limitations of BTM technique is its relay node selection process which is purely based on the distance between sender, receiver and the sink node. In EEBET, it is assumed that all the sensor nodes know their location information along with their depth. Depth information helps to select node towards sink and eliminate multiple hops. It also helps in eliminating loop formation. Depth threshold is incorporated to restrain number of nodes to become forwarder and select a node near optimum transmission range. Initially, all the nodes share their depth along with location information with each other. A node selects all nodes within its transmission range which are placed at lower depth than itself. Then the depth based selected nodes are further passed through the process of distance based calculation. The $N_{j}$ values for depth based neighbors are calculated using Equation (8). However, we need to choose only one relay node. Therefore, we also implement the process of distance based calculation to select only node at optimum transmission distance. A node selects only one node as a relay node, however, it maintains information of all of its neighbors along with their depth, $N_{j}$ values and energy information in its routing table. This is required to reselect the relay node incase of decrease in the RL of a node. This process continues for all the nodes to select their potential relay node. The list of nodes is sorted on the basis of $N_{j}$ values with minimum $N_{j}$ node placed at the top of the list. In order to eliminate number of hops involved in data transmission. Another contribution is selection of relay node in alternate ring sector rather than the neighbouring ring sector. A node in $R_{1}$ selects its relay node in $R_{3}$ while an $R_{2}$ node selects its relay in $R_{4}$. In this way, the data load of nodes is shared among different nodes and overall network energy is balanced. Route establishment in both schemes, EBET and EEBET is shown in Algorithm 2. The route establishment in EEBET differs EBET, as it considers depth of sensor nodes and calculates their depth difference, which is further compared against depth threshold. The EBET only follows steps 10–15 defined in Algorithm 2 after receiving $Q_{p}$.

**Algorithm 2:**: Route establishment in EEBET1:$Q_{p}$ ← Query packet2:$d_{th}$ ← Depth threshold3:$D_{sen}$ ← Depth of sender node4:$D_{rec}$ ← Depth of receiver node5:$D_{diff}$ ← Depth difference b/w sender and receiver node6:RNT ← Relay Node Table7:*Upon receiving* $Q_{p}$8:**if** $D_{rec}<D_{sen}$ **then**9: **if** $D_{diff}>d_{th}$ **then**10:  *Compute* $N_{j}$ *according to Equation eq:e8*11:  **if** $N_{j}=min\left(N_{j}\right)$ **then**12:   Add node ID in RNT13:  **else**14:   *drop* $Q_{p}$15:  **end** **if**16: **end** **if**17:**end** **if**

In BTM and EBET, nodes find their relays according to Equation (8). However, if there is a single node in the transmission range of a sender, it selects it as a relay node even if it is at higher depth than the sender. Also, the only receiver node may again select the same sender node as a forwarder which causes transmission loops. In this process, energy is consumed but the data packet cannot arrive at sink. If the high depth receiver node has its neighbor nodes and selects a node for data transmission other than the node from which it has received data packet, the hops are increased in number and energy dissipation occurs. We have proposed a solution for this problem which is discussed as follows.

##### Loop Detection and Elimination

**Figure 7:**
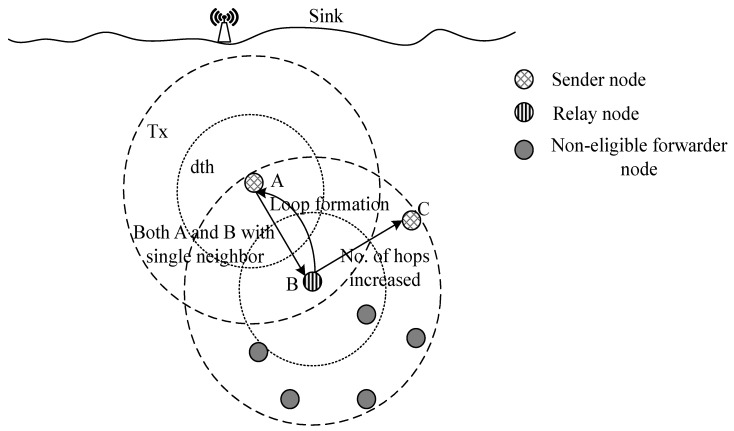
Formation of loops during data transmissions.

If a node has single neighbor node at higher depth than itself as shown in Figure 7, it is not selected as a relay node in EEBET. While BTM and EBET select it as a relay node which may cause transmission loop or result in extra number of hops, ultimately, energy get wasted. EEBET defines criteria for eliminating such loops and energy wastage by adjusting its transmission range accordingly [4]. It consumes more energy to transmit data at long distance, however, this occurs only for no or single neighbor node.

##### Data Transmission

During data transmission phase, nodes send data to their intended relay nodes. The initial energy of each sensor node is divided into Energy Levels. An Energy Level is calculated using Equation (9) while total optimum number of Energy Levels is calculated using Equation (29). As the data transmission process starts, each node transmits data to its selected relay node till its one energy level drops. When the RL of receiver node drops, it informs its receiver using $RLC_{p}$. The sender node again asks for the updated RL information from its pre-selected neighbor nodes in its routing table and selects a node with high residual energy than itself. The selected node receives data till its RL drops to next level. In this way all the network nodes consume almost same amount of energy and the network becomes energy balanced.

Consider four nodes A, B, C and D, where $A\in R_{1},B\in R_{2},C\in R_{3}$ and $D\in R_{4}$. Node *A* transmits its data packet to node *C* while node *B* transmit data to *D*. Nodes *A* and *B* transmit same amount of data at almost same distance, therefore, they consume same amount of energy. Node *C* receives data of *A* and also transmit its own data, than its unit energy level drops before node *A* consumes one energy level and informs its sender to reselect its relay node. Meanwhile, node *C* only send its own data packets. In this way, the data load is shared among different nodes and the node *A* starts transmitting its data to another node having high energy than itself. Therefore, it consumes its energy before another selected relay node drop one Energy Level. As a result, the energy becomes balanced between all Ring Sectors and the network becomes energy balanced.

Besides our contribution towards balanced energy transmissions, both EBET and EEBET pose the challenge of high packet drop rate. Therefore, we present linear programming based model to improve our EBET and EEBET techniques in terms of throughput. Details are given in the upcoming subsection.

### 4.2. Throughput Maximization Model

We use linear programming to design objective function and its constraints for throughput maximization as follows.

Objective function:

(30) $$Maximize\;\sum_{t=1}^{t_{max}}T_{p}\left(r\right)$$

Subject to:

(31a) $$E_{u}\leq E_{o}\;\forall \;u\in N$$

(31b) $$d_{u,v}\leq d_{opt}\;\forall \;u,v\in N$$

(31c) $$f_{u,v}\leq f_{max}\;\forall \;u,v\in N$$

(31d) $$d_{min}\leq d_{u}\leq d_{max}$$

(31e) $$P_{l}\geq P_{g}\;\forall \;u\in N$$

(31f) $$\begin{array}{c} \sum_{u=1}^{n}E\left(u\right)≅\sum_{v=1}^{n}E\left(v\right)\\ \forall \;n\in N,u=1-S_{r}\;and\;v=1-S_{r} \end{array}$$

Our objective in Equation (30) is to maximize the total number of successfully received packets at sink $T_{p}$ time span $t_{max}$ for which the network is alive. Equation (31a) deals with energy of sensor nodes; each sensor node *u* that belongs to the set of nodes *N* has limited battery supply with an upper bound $E_{o}$. Therefore, energy of sensor nodes should be utilized in an efficient way to prolong network lifetime, which in turn increases throughput. Equation (31b) means that the transmission distance between any two communicating nodes $d_{u,v}$ should be less than or equal to the optimum transmission distance $d_{opt}$. Failing to implement this constraint increases packet drop rate which means decreased throughput. Equation (31c) describes flow constraint of the physical link; the data flow between node *u* and *v* must be less than the upper bound $f_{max}$. Violation of this constraint leads to overflow situation leading to high packet drop rate. Equation (31d) illustrates the transmission distance of any node *u* from set *N*, $d_{u}$ must be optimum, provided the upper $d_{max}$ and lower bounds $d_{min}$. Transmitting data at very long distance by increasing transmission range leads to huge amount of packet drop. While transmitting at very short distance by decreasing transmission range leads to high energy consumption, hence, short network lifetime and decreased throughput. Our balanced energy transmission technique effectively compensated for this trade-off. Equation (31e) [24] suggests that the probability $P_{l}$ of current link status have to be at least $P_{g}$; *i.e.*, the minimum probability required for successful data delivery. Equation (31f) indicates that the total amount of energy consumed by all nodes in a ring sector needs to be approximately equal to any other ring sector node’s energy consumption. Network energy consumption is needed to be balanced in order to get high throughput. As long as the network is energy balanced, network remains alive and throughput increases. Therefore, to get high throughput, energy of all sensor nodes is required to be used in a balanced way.

## 5. Performance Evaluation

In this section, we evaluate the performance of our proposed protocols via simulations, and compare these with two existing protocols; BTM and UWDAR. The sensor nodes are deployed in a random uniform manner in a circular field with varying radius and sink at the center of the field. Except EEBET, each Ring Sector of the network field has equal number of sensor nodes such that these nodes are randomly deployed within the given ring sector. The network radius is denoted by R. The initial energy of each node is 300 J. Each sensor node generates a packet of 200 bits; 50 bits of control field and 150 bits of data field. For transmitting packets (channel access), the sensor nodes use Carrier Sense Multiple Access with Collision Avoidance (CSMA/CA) under the non-beacon enabled mode of IEEE 802.15.4. We have used Linprog linear programming solver in MATLAB to solve the optimization problem of throughput maximization. We have used three metrics for evaluating the performance of our proposed routing protocols; network lifetime, throughput, and path loss. The simulation parameters are shown in Table 3.

### 5.1. Impact of Varying Network Radius on Network Lifetime

**Figure 8:**
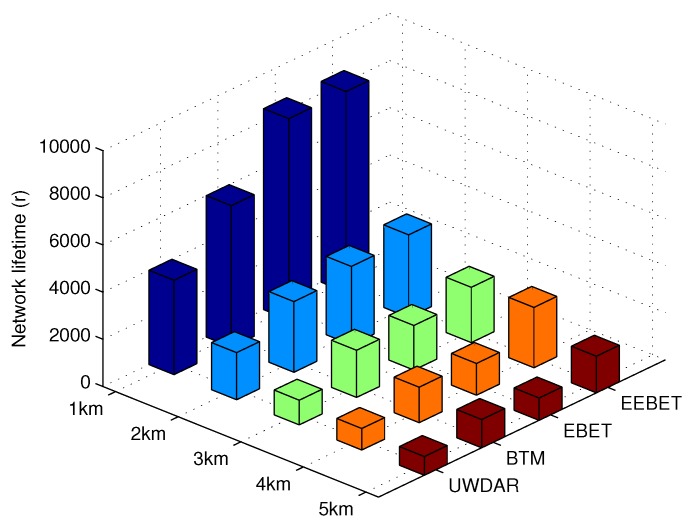
Comparison of network lifetime at different network radii.

The network lifetime of EEBET, EBET, BTM and UWDAR is shown in Figure 8. These simulation results are obtained for different network radii (*R*); ranging from 1 km to 5 km. From this figure it is clear that EEBET technique performs better than its counterpart techniques in terms of network lifetime. The UWDAR and BTM techniques show relatively shorter network lifetime at each network radius as compared to EBET and EEBET, respectively. BTM’s short network lifetime is due to excessive transmit energy consumption of farthest nodes that directly transmit data to the sink. UWDAR has very shorter network lifetime at every network radius due to backward data transmissions. The sensor nodes nearer to sink, except the first hop-grade nodes, transmit data to far-away in-charge hop-grade nodes. Thus, the nodes at long distance from sink quickly deplete their energy and leading to network partitioning. The EBET technique has improved network lifetime when compared to BTM. The reason for its network lifetime improvement is the exclusion of one hop transmissions over long distance. However, this leads to increased number of hops in data transmission. Also, the energy consumption in both BTM and EBET is balanced between successive Ring Sectors, whereas, the overall network energy consumption is not balanced. The maximum difference of Energy Levels between successive Ring Sectors is one for small scale UWSNs in BTM and large scale UWSNs in EBET. The nodes nearer to the sink completely deplete their energy while the other nodes in the network are left with residual energy which leads to energy wastage. EEBET improves network lifetime due to two reasons; (i) minimized number of hops involved in data transmission; and (ii) reduced data load on nodes nearer to the data sink. When the distance between sensor nodes and sink decreases, their data load is managed. Therefore, overall its network lifetime is improved.

### 5.2. Impact of Varying Network Radius on Transmission Loss

**Figure 9:**
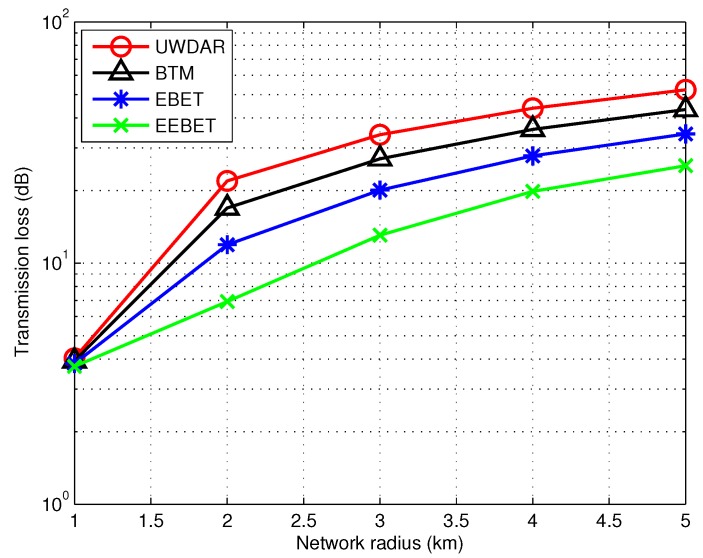
Comparison of transmission loss at different network radii.

Transmission loss for EEBET, EBET, BTM and the UWDAR techniques is shown in Figure 9. Form this figure, it is clear that the transmission loss of BTM and UWDAR is relatively high as compared to EBET and EEBET. Since transmission loss depends on spreading loss and distance, both BTM and UWDAR schemes use direct transmissions which leads to high path loss. The EBET and the EEBET minimize the communication distance to achieve relatively low transmission loss. This figure also shows the impact of network radius increase on the transmission loss. In this regard, EBET and EEBET exhibit less increase whereas BTM and UWDAR show very high increase. Thus, our proposed techniques are less affected by the network radius as compared to the selected existing ones.

### 5.3. Impact of Varying Network Radius on Throughput

**Figure 10:**
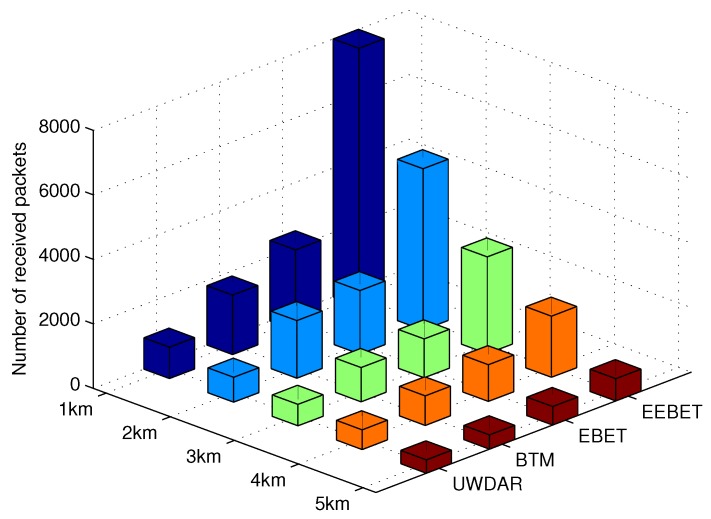
Comparison of throughput at differen network radii.

The total number of packets received during network lifetime is shown in Figure 10. EEBET has improved throughput than EBET, BTM and UWDAR. In EEBET, the network remains alive for relatively longer duration of time for the reception of data packets at sink as compared to EBET, BTM and UWDAR. In EBET, BTM and UWDAR, the sensor nodes deplete their energy at an earlier stage as compared to EEBET. Thus, leading to network partitioning in very short time. Therefore, their throughput is less than the EEBET. The EBET has high throughput as compared to BTM and UWDAR due to increased number of hops involved in data transmission. All the sensor nodes transmit data only to their one hop neighbor nodes, whereas in BTM, a distant node’s direct data transmission to the sink consumes more than one energy level in sending one data packet. So, the node depletes energy earlier and network collapses. Hence no more packets can be received. Also, direct data transmission at long distance face high attenuation that increases the chances of packet(s) being dropped. BTM’s direct data transmission leads to high number of dropped packets, consequently, low throughput is observed in Figure 10. The UWDAR also considers direct data transmissions for in-charge hop-grade nodes. Therefore, its throughput is also less than BTM, EBET and EEBET. The involvement of backward data transmissions in UWDAR leads to highly imbalanced energy consumption, where few nodes consume very high energy while maximum nodes have high residual energy. As a result, the sensors nodes die within short time, hindering successful packet reception at sink. Similar to the lifetime, the network throughput of all schemes decreases with increasing network radius due to rapid energy levels reduction and sensor nodes energy depletion. Thus, less throughput is observed in Figure 10 while increasing network radius.

### 5.4. Impact of Varying Number of Nodes and Varying Network Radius on Network Lifetime

**Figure 11:**
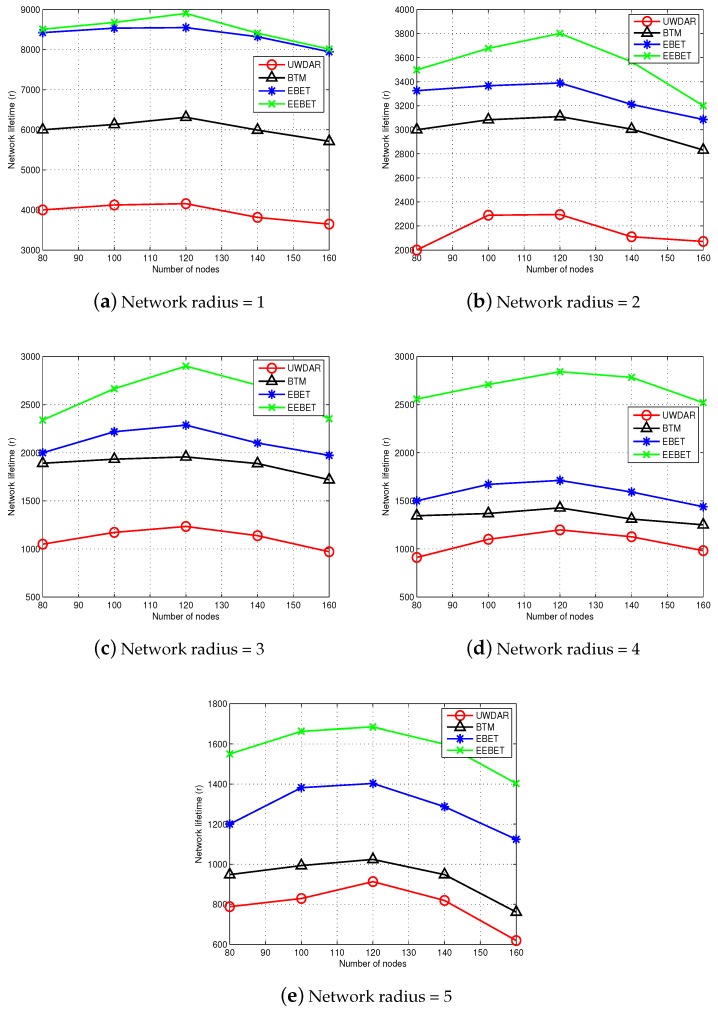
Impact of number of nodes and network radius on network lifetime.

We have simulated EEBET, EBET, BTM and UWDAR with varying number of nodes in the network field of different radii to check the scalability of the existing and the proposed schemes in terms of network lifetime. Figure 11a–e shows the results for network radius ranging from 1 km to 5 km such that the number of nodes vary from 80 to 160. It is evident from the figure that both EEBET and EBET’s lifetime first increases and then decreases when the number of nodes are increased from 80 to 160. Moreover, when the number of nodes exceeds 120, network lifetime of EEBET, EBET and BTM exhibit slight decrease. Also, both EBET and EEBET have longer network lifetime than BTM and UWDAR protocols with increasing number of nodes. All the schemes face decay in network lifetime with increasing number of nodes. This is due to increased congestion and interference when the number of nodes are increased. However, EEBET’s performance is not affected in high proportion, by increasing the number of nodes in network. This is because of the global energy balancing in EEBET which which makes this technique more scalable.

### 5.5. Impact of Varying Number of Nodes and Varying Network Radius on Throughput

**Figure 12:**
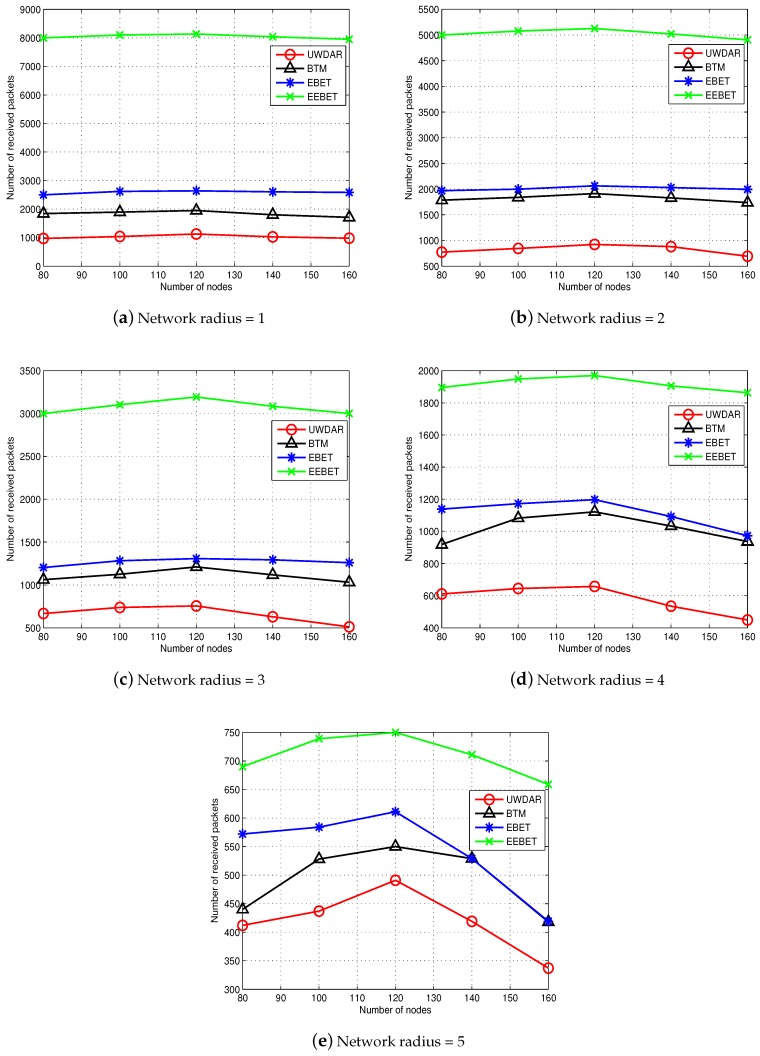
Impact of number of nodes and network radius on throughput.

Figure 12a–e shows the number of received packets at sink while implementing UWDAR, BTM, EBET and EEBET, respectively. These results are obtained at different network radii (ranging from 1 km to 5 km) while varying the number of nodes between 80 and 160 for each network radius. In these results, initially, the packet reception rate shows an increasing behaviour and then a decreasing behaviour. The increasing behaviour is due the added number of nodes in the network field as we go from 80 to 100 nodes. From 100 to 160 nodes, the packet reception rate decreases because the added nodes contend for channel to deliver their packets to the sink. This scenario results in increased congestion at the sink which increases the chances of packets’ collision at the sink and high packet collision rate means decreased number successfully received packets at the sink. It is evident from Figure 12a–e that our proposed protocols (EBET and EEBET) show better performance than the existing ones (BTM and UWDAR) due balanced transmission mechanism and linear programming based optimization. The EEBET is more scalable than the rest of the protocols due to global balanced transmissions.

## 6. Conclusions

In this paper, we have analyzed energy consumption of UASNs and investigated reasons of rapid energy depletion. We have proposed two new routing protocols, EBET and EEBET. These protocols overcome the energy inefficiency and unbalancing deficiencies in existing UASN routing protocols. The use of Energy Levels help in balanced energy consumption of sensor nodes. The techniques are proved to be efficient for large scale UASNs in terms of network lifetime prolongation and throughput maximization. The EEBET technique improved the energy balancing and increased the network lifetime. The EEBET incorporates depth threshold to limit the number of nodes towards sink and eradicate backward data transmissions. We evaluated the performance of our proposed protocols by comparing them with BTM and UWDAR in terms of network lifetime, transmission loss, and throughput. We also checked the relative scalability of our protocols by varying the number of nodes and the network radius. Simulation results show the improved performance of our proposed techniques than their counterpart schemes in terms of the selected performance metrics.

## Figures and Tables

**Table 1:** Comparison of the state-of-the-art work.

Technique	Features	Domain	Flaws/Deficiencies	Achievements
BTM [9]	Balanced energy consumption, Energy efficient	UASNs, 2D underwater terrain monitoring	High energy consumption while transmitting at long distance, Formation of transmission loops	Network lifetime, Balanced energy consumption
EBH and DIB [3]	Energy balancing, Applicable for both shallow and deep water	2D UASNs, Moored monitoring systems, Oceanographic data collection	Inefficient for other than linear networks, Designed only for sparse network	Network lifetime, Balanced energy consumption
H2-DAB [8]	Energy efficient, Dimensional location information is not required	UASNs, Critical underwater monitoring missions	Imbalanced energy consumption, Nearer to sink nodes deplete energy earlier, High end-to-end delay	High data delivery ratio, Network lifetime
RAD [15]	Energy efficient, Acoustic channel utilization efficiency, Introduces forward error correction	3D UASNs, Geographical routing for delay-sensitive and delay-insensitive applications	Imbalanced energy consumption, Increase in packet size increases packet error rate, Solution for concave node (void region) is not provided	Low packet error rate, Minimized energy consumption, Low end-to-end delay
ERP2R [16]	Energy efficient, Physical distance and residual energy based routing	3D UASNs, Underwater monitoring, Location-based routing	Imbalanced energy consumption with the growth of node mobility, Longer routing paths, Duplicate packets transmission	Network lifetime, Minimized end-to-end delay and energy consumption
RDBF [11]	Energy efficient, Minimum hop counts involved, No exchange of control messages	3D UASNs, Underwater monitoring and target tracking applications, Location-based routing	Do not restrict forwarding area for packets, All nodes with same distance with the sink send same packets at the same time	Network lifetime, High packet delivery ratio, Low end-to-end delay
DBR [6]	Full dimensional location information of sensor nodes is not required	UASNs, 3D underwater monitoring, Depth based routing, Handles dynamic networks	High energy consumption, Imbalanced energy consumption, Data redundancy, Inefficient for sparse and highly dense networks	Network lifetime, High data delivery ratio, Low end-to-end delay
EEDBR [5]	Energy efficient, No dimensional location information required, Controlled flooding	UASNs, 3D underwater monitoring and surveillance applications	Imbalanced energy consumption, High energy consumption in dense networks, Low packet delivery ratio, High packet drop rate	Network lifetime, Minimum energy consumption, End-to-end delay
UFCA [7]	Energy efficient, No periodic flooding messages required, Uses Gravity function for data routing	3D-UASNs, Underwater surveillance and monitoring applications, Distributed routing mechanism	Imbalanced energy consumption, Resistant to node mobility, Temporary loss of connectivity, Routing information may not be updated, High end-to-end delay	High packet delivery ratio, Minimized energy consumption, Scalability
CDBR/CEEDBR [17]	Energy efficient, Limited forwarder nodes, Depth-dependent	3D-UASNs, Underwater monitoring and surveillance	Imbalanced energy consumption, High packet drop and end-to-end delay, Static network topology	Network lifetime, Minimized energy consumption

**Table 2:** Optimal threshold against network radius.

$O_{t}$ (km)	R (km)
0.15	1
0.225	1.5
0.3	2
0.375	2.5
0.45	3
0.525	3.5
0.6	4
0.675	4.5
0.75	5

**Table 3:** Simulation parameters.

Parameter	Value
*R*	1–5 km
*N*	80
$E_{0}$	300 J
*f*	20 kHz
$P_{r}$	0.2 × $10^{-4}$ J/bit
